# Esketamine clinical trials: reply to Maju *et al.*

**DOI:** 10.1017/S204579602000027X

**Published:** 2020-04-29

**Authors:** C. Gastaldon, D. Papola, G. Ostuzzi, C. Barbui

**Affiliations:** Department of Neuroscience, Biomedicine and Movement Sciences, Section of Psychiatry, University of Verona, Verona, Italy

**Keywords:** Esketamine, evidence-based medicine, FDA, regulatory policies, treatment-resistant depression

## Abstract

Maju *et al.* provided clarifications on important and controversial issues related to esketamine clinical trial data, in response to a vivid debate triggered by the marketing authorisation recently granted by this new medicine. In this commentary, we reply to their comments attempting to critically discuss the evidence base needed to obtain regulatory approval.

We cordially thank Maju *et al.* and colleagues for providing clarifications on important and controversial issues related to esketamine clinical trial data (Gastaldon *et al*., 2020; Maju *et al*., 2020). These clarifications are crucial in light of the vivid debate triggered by the marketing authorisation recently granted by this new medicine.

One of the core aspects of this debate derives from the definition of clinical significance threshold. Maju *et al.* commented that the difference reported between esketamine and placebo in the only positive esketamine short-term efficacy trial (TRANSFORM-2), subsequently confirmed by a meta-analysis of the three existing short-term efficacy trials (Gastaldon *et al*., 2020; Maju *et al*., 2020), is clinically significant. We kindly disagree with this interpretation. Indeed, the efficacy results of the TRANSFORM-2 trial (mean change = 4.4 MADRS points) and of the meta-analysis (mean change = 4.08 MADRS points; 95% confidence interval 1.99 to 6.18) are aligned, favouring esketamine over placebo. However, establishing a minimal clinically relevant difference is contentious (Naudet and Cristea, 2020), and the literature provides contradictory suggestions. Some authors suggested a threshold for minimal clinical significance of 2 MADRS points, whereas others considered a difference of at least 7–9 MADRS points as clinically significant (Duru and Fantino, 2008; Leucht *et al*., 2017). Ultimately, any threshold should be weighed considering the drug's tolerability and acceptability profile, as indicated by Naudet and Cristea (2020). As esketamine carries well-known risks, such as dissociation and potential of abuse (Schatzberg, 2019; Turner, 2019), we believe it is reasonable and cautious to consider a higher threshold compared to other less risky medications – as it was actually done in the TRANSFORM-2 trial by setting a threshold of 6.5 MADRS points for clinical significance, to be used for power estimations (Popova *et al*., 2019).

Maju *et al.* commented that the 6.5 MADRS threshold was not used for establishing a minimal clinically significant difference (Maju *et al*., 2020), but only for power estimation purposes. We have two comments on this. First, if 6.5 MADRS points do not reflect a minimal clinically significant difference than one wonders why it was used for the power calculation. Second, any positive result below this pre-set threshold carries a risk of being a false positive, as Naudet and Cristea recently pointed out (Naudet and Cristea, 2020). In general, the lower the power of a study, the lower the probability that a statistically significant observed finding (*p* < 0.05) actually reflects a true effect. Even when an underpowered study detects a true effect, the estimate of the effect magnitude may be exaggerated (Button *et al*., 2013). In policy-making, few things are less desirable than making decisions relying on irresolute evidence. For this reason, we suggested that the regulatory agencies should consider regulatory meta-analyses during the drug approval process (Barbui *et al*., 2017), as pooling results from various studies on the same research question may overcome the power limitation of individual studies.

Further, Maju *et al.* suggested that the difference observed at day 2 between esketamine and placebo is unlikely to be related to esketamine antidepressant effect. This seems very reasonable, as this rapid change may likely be related to a transient psychotropic effect induced by esketamine, and not to an actual and lasting recovery from depression (Moncrieff, 2018).

We previously commented that the FDA and EMA made approval decisions only considering four trials (TRANSFORM-1, -2, -3 and SUSTAIN-1) (Daly *et al*., 2019; Fedgchin *et al*., 2019; Popova *et al*., 2019; Gastaldon *et al*., 2020; Ochs-Ross *et al*., 2020), and that none of these studies provided long-term data. Maju *et al.* responded that in the meantime another trial was concluded, SUSTAIN-2. We argue, however, that the findings of SUSTAIN-2 were not available at the time of FDA and EMA evaluation, and therefore only short-term data were considered during the approval process. We believe that this is a major aspect, as regulatory decisions were taken regardless of long-term information on medicines that may have tolerability and safety risks.

Another point raised in our commentary, as well as in other similar comments (Cristea and Naudet, 2019; Schatzberg, 2019; Turner, 2019), refers to the need for further data to support esketamine benefits in maintenance treatment. Maju *et al.* reported that an additional trial was required by the FDA, since there was only one short-term efficacy trial with positive results at the time of FDA approval (Popova *et al*., 2019). Consequently, a withdrawal trial was designed in which participants achieving remission or response after 12–16 weeks of esketamine treatment were randomised to discontinue or continue esketamine (Daly *et al*., 2019). Patients randomised to discontinue esketamine showed higher relapse rates compared to patients continuing esketamine. We reason that this effect might be related either to esketamine efficacy (as claimed by Maju *et al.*) or to a detrimental potential of esketamine discontinuation. As a matter of fact, it cannot be excluded that withdrawal symptoms biased the outcome assessment (Turner, 2019). In particular, the abrupt interruption of esketamine might have contributed to depressive symptoms via withdrawal phenomena, raising doubts on the benefits of esketamine maintenance treatment (Schatzberg, 2019).

Maju *et al.* commented that the safety profile of esketamine is well known from phase 1 and 2 studies and that the drug is safe (Maju *et al*., 2020). Our meta-analysis, based on data submitted to FDA and EMA, detected a significantly worse acceptability profile of esketamine compared to placebo, with a higher proportion of participants dropping out due to any reasons (relative risk 1.63, 95% confidence interval 1.02–2.60, three studies, 711 participants, no heterogeneity) (Gastaldon *et al*., 2020). Similarly, the incidence of dissociation was seven times higher for participants taking esketamine in comparison with those taking placebo, with approximately 25% of esketamine-treated patients experiencing dissociation during treatment (Gastaldon *et al*., 2020). Although some authors hypothesised that the experience of dissociation might mediate the antidepressant effect, nonetheless it remains a severe adverse effect both in the short-term and in the long-term. In the long-term, additionally, other potentially serious adverse effects have been suggested, including even persistent schizophrenia-like symptoms after prolonged use (Molero *et al*., 2018, Chen *et al*., 2020).

Maju *et al.* claimed that the SUSTAIN-1 trial (Daly *et al*., 2019) provided long-term data (Maju *et al*., 2020). In our opinion, a trial with a follow-up of 2 weeks can hardly be considered a long-term study. Current guidelines suggest at least 6–8 months (24–32 weeks) of maintenance treatment for people with the first episode of major depression and longer treatment duration for patients with recurrent episodes (APA, 2010; NICE, 2018). It is therefore not surprising that patients of the SUSTAIN-1 trial discontinuing treatment after just 12 or 16 weeks of treatment showed more relapse episodes than those continuing treatment. Doctors still do not know for how long esketamine should be prescribed, and which long-term effects and side effects are to be expected. We argue that this lack of information is a real disservice to people suffering from depression.

In conclusion, considering the notable uncertainty on tolerability and efficacy, both in the short- and in long-term, we argue that regulatory authorities should have to be more cautious in evaluating the approval of this drug (Cristea and Naudet, 2019; Schatzberg, 2019). Now that esketamine is on the market, it is up to national medicine agencies to wisely regulate its use. In the UK, for example, the National Institute for health and Care Excellence (NICE) decided not to recommend esketamine for use in clinical practice based on similar considerations (NICE, 2020; Mahase, 2020). Waiting for new evidence on esketamine efficacy and tolerability, we hope that other national agencies will provide guidance based on a careful evaluation of currently available evidence.
